# Single-cell insights: pioneering an integrated atlas of chromatin accessibility and transcriptomic landscapes in diabetic cardiomyopathy

**DOI:** 10.1186/s12933-024-02233-y

**Published:** 2024-04-25

**Authors:** Qiang Su, Wanzhong Huang, Yuan Huang, Rixin Dai, Chen Chang, Qiu-Yan Li, Hao Liu, Zhenhao Li, Yuxiang Zhao, Qiang Wu, Di-Guang Pan

**Affiliations:** 1 Department of Cardiology, People’s Hospital of Guilin, Guilin, China; 2 Department of Cardiology, Jiangbin Hospital of Guangxi Zhuang Autonomous Region, Nanning, China; 3 Department of Cardiology, Affiliated Hospital of Guilin Medical University, Guilin, China; 4 Institute of Bioengineering, Biotrans Technology Co., LTD, Shanghai, China; 5 United New Drug Research and Development Center, Biotrans Technology Co., LTD, Changsha, China; 6 College of Pharmaceutical Sciences, Zhejiang University, Hangzhou, China; 7 BoYu Intelligent Health Innovation Laboratory, Hangzhou, China; 8 Senior Department of Cardiology, the Sixth Medical Centre, Chinese PLA General Hospital, Beijing, China

**Keywords:** DCM, scRNA-seq, scATAC-seq, Cardiomyocytes, Fibroblasts, Endothelial

## Abstract

**Background:**

Diabetic cardiomyopathy (DCM) poses a growing health threat, elevating heart failure risk in diabetic individuals. Understanding DCM is crucial, with fibroblasts and endothelial cells playing pivotal roles in driving myocardial fibrosis and contributing to cardiac dysfunction. Advances in Multimodal single-cell profiling, such as scRNA-seq and scATAC-seq, provide deeper insights into DCM’s unique cell states and molecular landscape for targeted therapeutic interventions.

**Methods:**

Single-cell RNA and ATAC data from 10x Multiome libraries were processed using Cell Ranger ARC v2.0.1. Gene expression and ATAC data underwent Seurat and Signac filtration. Differential gene expression and accessible chromatin regions were identified. Transcription factor activity was estimated with chromVAR, and Cis-coaccessibility networks were calculated using Cicero. Coaccessibility connections were compared to the GeneHancer database. Gene Ontology analysis, biological process scoring, cell-cell communication analysis, and gene-motif correlation was performed to reveal intricate molecular changes. Immunofluorescent staining utilized various antibodies on paraffin-embedded tissues to verify the findings.

**Results:**

This study integrated scRNA-seq and scATAC-seq data obtained from hearts of WT and DCM mice, elucidating molecular changes at the single-cell level throughout the diabetic cardiomyopathy progression. Robust and accurate clustering analysis of the integrated data revealed altered cell proportions, showcasing decreased endothelial cells and macrophages, coupled with increased fibroblasts and myocardial cells in the DCM group, indicating enhanced fibrosis and endothelial damage. Chromatin accessibility analysis unveiled unique patterns in cell types, with heightened transcriptional activity in myocardial cells. Subpopulation analysis highlighted distinct changes in cardiomyocytes and fibroblasts, emphasizing pathways related to fatty acid metabolism and cardiac contraction. Fibroblast-centered communication analysis identified interactions with endothelial cells, implicating VEGF receptors. Endothelial cell subpopulations exhibited altered gene expressions, emphasizing contraction and growth-related pathways. Candidate regulators, including Tcf21, Arnt, Stat5a, and Stat5b, were identified, suggesting their pivotal roles in DCM development. Immunofluorescence staining validated marker genes of cell subpopulations, confirming PDK4, PPARγ and Tpm1 as markers for metabolic pattern-altered cardiomyocytes, activated fibroblasts and endothelial cells with compromised proliferation.

**Conclusion:**

Our integrated scRNA-seq and scATAC-seq analysis unveils intricate cell states and molecular alterations in diabetic cardiomyopathy. Identified cell type-specific changes, transcription factors, and marker genes offer valuable insights. The study sheds light on potential therapeutic targets for DCM.

**Supplementary Information:**

The online version contains supplementary material available at 10.1186/s12933-024-02233-y.

## Graphical Abstract

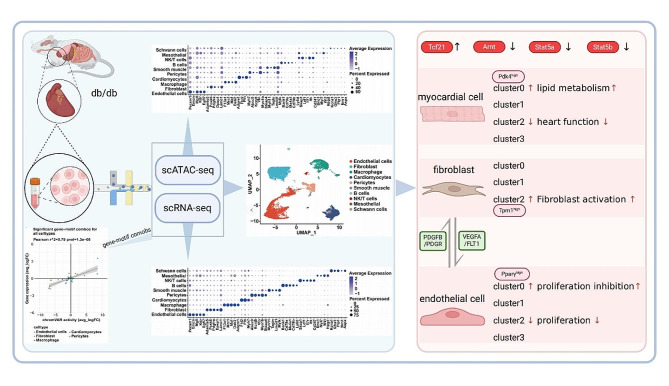

## Introduction

Diabetic cardiomyopathy (DCM) stands as a critical health concern with increasing global prevalence [1]. Despite lacking overt cardiovascular issues, individuals with diabetes face a heightened risk of developing heart failure due to DCM. In 2021, an alarming 536 million people worldwide aged 20 to 79 were affected by diabetes, comprising 10.5% of this age group [2]. The incidence of heart failure in diabetic patients, ranging from 13 to 30% [3, 4], emphasizes the urgent need to comprehend and address the clinical implications of DCM. Unraveling the complexities of DCM is imperative for developing targeted interventions that could mitigate the progression of heart failure in diabetic individuals.

Understanding the intricate cellular mechanisms behind DCM involves a focus on fibroblasts and endothelial cells, two key players in cardiac function and pathology. Myocardial fibrosis, a hallmark of DCM, is driven by the transformation of cardiac fibroblasts into active myofibroblasts. These cells contribute to the excessive deposition of extracellular matrix (ECM) proteins, disrupting normal cardiac function and leading to cardiac remodeling and even heart failure [5, 6]. Additionally, endothelial cells undergo endothelial-mesenchymal transition (EndMT) during diabetes, further contributing to fibrosis [7–9]. Investigating the interactions between fibroblasts, endothelial cells, and other cardiac cell types is crucial to deciphering the molecular intricacies of DCM [10]. Insights gained from these cellular interactions could open avenues for targeted therapeutic strategies aimed at preventing or reversing myocardial fibrosis.

Advancements in technology have revolutionized our ability to unravel the molecular underpinnings of DCM. Single-cell RNA sequencing (scRNA-seq) and single-cell Assay for Transposase-Accessible Chromatin sequencing (scATAC-seq) have emerged as key technologies. scRNA-seq provides a detailed look at gene expression at the individual cell level, offering insights into the dynamic changes within various cell populations in response to diabetes-induced cardiac pathology. This technique has been particularly illuminating in understanding the transcriptomic alterations in fibroblasts, endothelial cells, myocardial cells, and macrophages in a diabetic cardiomyopathy mouse model [11]. On the other hand, scATAC-seq complements scRNA-seq by providing information on chromatin accessibility, shedding light on the regulatory elements controlling gene expression [12]. Together, these technologies could offer a comprehensive view of the molecular landscape of DCM, facilitating the identification of potential therapeutic targets and pathways for intervention. Harnessing the power of these advanced techniques is integral to mining the intricate details of disease development in DCM and holds promise for developing more effective treatment strategies.

## Method

### Animals

Because db/db mice (C57BLKS/J background) are close to the disease mechanism and pathological phenotype of human type 2 diabetes, it is widely used in the scientific research of diabetes cardiomyopathy. 22 12-week-aged db/db male mice were used as DCM group, and 16 12-week-aged db/m male mice were used as wide type (WT) group. After the model were built, 10 mice cardiac tissues was harvested from each group and underwent careful processing to isolate individual cells for single-cell transcriptome and chromatin accessibility sequencing, the other mice cardiac tissues were used for immunofluorescence assay. Animal experiments were approved by the Animal Ethics Committee of Guilin Medical University (No. GLMC201905012) and followed the National Institutes of Health Guidelines on the Care and Use of Animals.

### 10x Multiome (RNA + ATAC) library preparation and data processing

Raw single-cell sequencing data were analyzed using the Cell Ranger ARC v2.0.1 pipeline (10X Genomics). Reads were aligned to the mouse genome reference (mm10).

### Quantification, quality control, and cluster analysis of single-cell RNA and ATAC data

For gene expression data, the cells that expressed less than 200 or more than 6000 unique genes, or more than 10% of reads mapping to mitochondria, or more than 0.5% of reads mapping to hemoglobin were filtered out by the Seurat R package (version 4.2.0) [13]. For ATAC data, the cells that detected less than 1000 or more than 100,000 ATAC reads were filtered out by the Signac R package (version 1.8.0) [14]. Then, we only reserved the cells with lower nucleosome signal (< 1) and higher TSS enrichment (> 1). Cells presumed as doublets by DoubletFinder [15] R package (version 2.0.3) were filtered out. Then, we merged all samples and performed peak calling using MACS2 (2.2.7.1 version) [16] with default parameters. We processed the data for two modalities separately. For gene expression data, the global scaling method was used for normalizing, and the function ScaleData was used for removing unwanted sources of variation. In addition, principal component analysis (PCA) was used for calculating the most significant 20 principal components (PCs) of the gene expression.

For ATAC data, we only reserved peaks that were detected in more than ten cells. Term frequency-inverse document frequency (TF-IDF) was used for normalizing and singular value decomposition (SVD) was used for dimensional reduction. Then, we integrated all samples with anchors. Specifically, we found a set of anchors between two datasets using the function FindIntegrationAnchors and integrated them using the IntegrateData. To integrate information from multiple modalities, a weighted nearest neighbor (WNN) graph [17] was constructed according to the integrated dimensional reductions from two modalities. Next, the function RunUMAP was used for Uniform Manifold Approximation and Projection (UMAP) dimensional reduction, and the function FindClusters was used to cluster cells. The expression of known marker genes was used to annotate each cluster.

For gene expression data, the list of differential genes between cell types was calculated with function FindMarkers in Seurat and filtered by following settings (min.pct = 0.2, logfc.threshold = 0.2, only.pos = TRUE). Adjusted *p*-value (Wilcoxon test) was used to determine significance at an FDR < 0.05.

For ATAC data, the list of differential accessible chromatin regions (DAR) between cell types was calculated with the FindMarkers function in Signac and filtered by following settings (min.pct = 0.2, logfc.threshold = 0.2, only.pos = TRUE). Adjusted *p*-value calculated by logistic regression framework (LR) was used to determine significance at a FDR < 0.05.

### Transcription factor activity estimation

Transcription factor activity was estimated using the ATAC profile with chromVAR (version 1.16.0) [18]. JASPAR2018 database provided the positional weight matrix during the calculation. By taking advantage of the function RunChromVAR wrapper in Signac, Cell-type-specific chromVAR activities were calculated. The list of differential activity between cell types was calculated with the FindMarkers function in Signac and filtered by adjusted *p*-value (FDR < 0.05). The function FindMotif was also used for motif enrichment analysis on differentially accessible regions.

### Cis-coaccessibility networks calculation with Cicero

Cis-coaccessibility networks were calculated using the ATAC profile with Cicero (version 1.12.0) [19]. The data for each cell type was converted to cell dataset (CDS) objects by function make_atac_cds separately. After processing by function detect_genes and estimate_size_factors, dimensionality reduction analysis was performed on each CDS object and all CDS objects were converted to Cicero CDS object. The function run_cicero calculated the cell-type-specific Cicero connections.

### Comparison of Cicero coaccessibility connections to GeneHancer database

The list of differentially accessible chromatin regions (DAR) of different cell types was filtered by following settings (min.pct = 0.2, logfc.threshold = 0.2, only.pos = TRUE, adjusted *p*-value < 0.05). All the Cell-type-specific DAR were extended up and down 50 kb and the top 1000 DAR by log fold change were used to create bed files. Files were transferred to GeneHancer [20] interaction tracks on the UCSC table browser. The mean proportion of overlap between GeneHancer interactions and cell-type-specific Cicero connections was compared with the Cicero co-access threshold.

### Gene ontology enrichment analysis

The FindAllMarkers function was used to calculate the differential genes of each cell subset by comparing the cell subset with other cells. To do the further analysis, the list of differential genes filtered by following settings (only.pos = TRUE, min.pct = 0.2, logfc.threshold = 0.2) and the list of differential genes at an adjusted *p*-value (Wilcoxon test) < 0.05 were retained. We selected the top 100 genes in fold change to perform Gene Ontology (GO) enrichment analysis using clusterProfiler R package [21]. The results of the enrichment analysis were selected based on the statistical threshold (qvalueCutoff = 0.05) and the results belonging to Biological Process (BP) were reserved.

The list of differential genes of each cell subset by comparing the cell subset with other cells was calculated by function FindAllMarkers and was filtered by following settings (only.pos = TRUE, min.pct = 0.2, logfc.threshold = 0.2). Adjusted *p*-value (Wilcoxon test) was used to determine significance at an FDR < 0.05. The top 100 genes in fold change were selected to perform Gene Ontology (GO) enrichment analysis with clusterProfiler R package (version 4.2.2). We concentrated on enrichment analysis results belonging to Biological Process (BP) and they were selected based on the statistical threshold (qvalueCutoff = 0.05).

### Scoring of biological processes

The scores of individual cells were generated according to the gene signatures representing biological functions, and they were defined as the average normalized expression of corresponding genes. Functional signatures were collected from the Gene Ontology database [22], and they are all differential genes at an adjusted *p*-value cut-off of 0.05 using the Wilcoxon test.

The scores of individual cells were determined using the average normalized expression of the gene in question. These genes, or gene signatures, represent corresponding biological functions, which makes the score a measure of the biological processes of each cell. These functional signatures are both differential genes (adjusted *p*-value < 0.05) and at the same time genes in the Gene Ontology database.

### Cell-cell communications analysis

The top 10,000 variables were selected, and the genes expressed in less than ten cells were filtered out. Mouse genes were converted to human paralogues with the GRCm38/GRCh38 genome reference. Cell-cell communications analysis was performed using CellphoneDB (version 3.1.0) [23] based on the expression matrix. In the results of CellPhoneDB, the ligand-receptor pairs with no valid values were filtered out.

### Gene-motif correlation analysis

The mean value of cell-specific motif activity was first calculated by chromVAR, and we correlated this activity with the corresponding gene expression using log(fold change).

### Immunofluorescent staining

Sections of paraffin-embedded tissues were deparaffinized and underwent antigen retrieval. Sections were blocked with 1% bovine serum albumin, permeabilized with 0.1% Triton-X100 in PBS and incubated overnight with primary antibodies for cTnT (abcam), Pdk4 (proteintech), Vimentin (Cell Signaling Technology), Tpm1 (Invitrogen), CD31 (abcam), or Pparg (proteintech). These sections were subsequently stained with secondary antibodies, Alexa Fluor 488 goat anti-rabbit antibody (abcam), Cy3 goat anti-rabbit antibody (abcam); After sections were stained with DAPI (4’, 6’-diamidino-2-phenylindole), images were obtained by confocal microscope.

### Statistic Analysis

The statistical analysis of all data in this study was conducted using the respective R software packages, and a significance level of *P* < 0.05 was considered statistically significant.

## Results

### Cell type change during the DCM progression

**Fig. 1:**
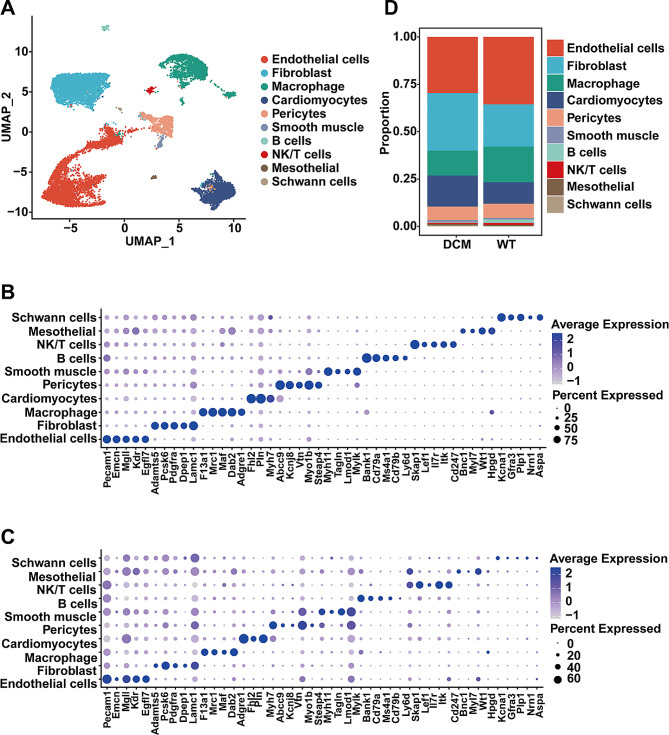
Cell types in DCM and WT hearts. **A**. UMAP plot of multi-omics dataset. Cell types are annotated by marker genes. **B**. Bubble plot of cell-type-specific marker genes in RNA profile. The diameter of the dot represents the proportion of cells that express the corresponding gene and the density of the dot represents the average gene expression level among all cell types. **C**. Bubble plot of Cell-type-specific marker gene activity inferred from scATAC data. The diameter of the dot represents the proportion of cells that express the corresponding gene and the density of the dot represents the average gene expression level among all cell types. **D**. Comparison of relative proportions of cell types between WT and DCM groups

In this study, we integrated and dimensionality-reduced the single-cell RNA sequencing (scRNA-seq) and single-cell ATAC sequencing (scATAC-seq) data from WT (wild type) and DCM (diabetic cardiomyopathy) mouse models. Subsequently, we performed clustering analysis on the integrated data, annotated the cell populations using classical markers, and identified distinct cell clusters, including endothelial cells and fibroblasts (Fig. 1A). To validate the accuracy and reliability of our clustering, we employed bubble plots to illustrate the expression profiles of different cell markers within the defined cell types for both scRNA-seq and scATAC-seq data (Fig. 1B and C). The results indicate that our clustering approach is robust and accurate. Furthermore, by comparing the proportions of cell types between WT and DCM mouse models, we observed a decrease in the proportion of endothelial cells and macrophages, while fibroblasts and myocardial cells exhibited an increase in abundance in the DCM mouse group (Fig. 1D) when compared to the WT group, indicating increased fibrosis and endothelial damage during the DCM progression, consistent with previous reports [24, 25].

### Cell type-specific chromatin accessible regions and cell cycle change during DCM

**Fig. 2:**
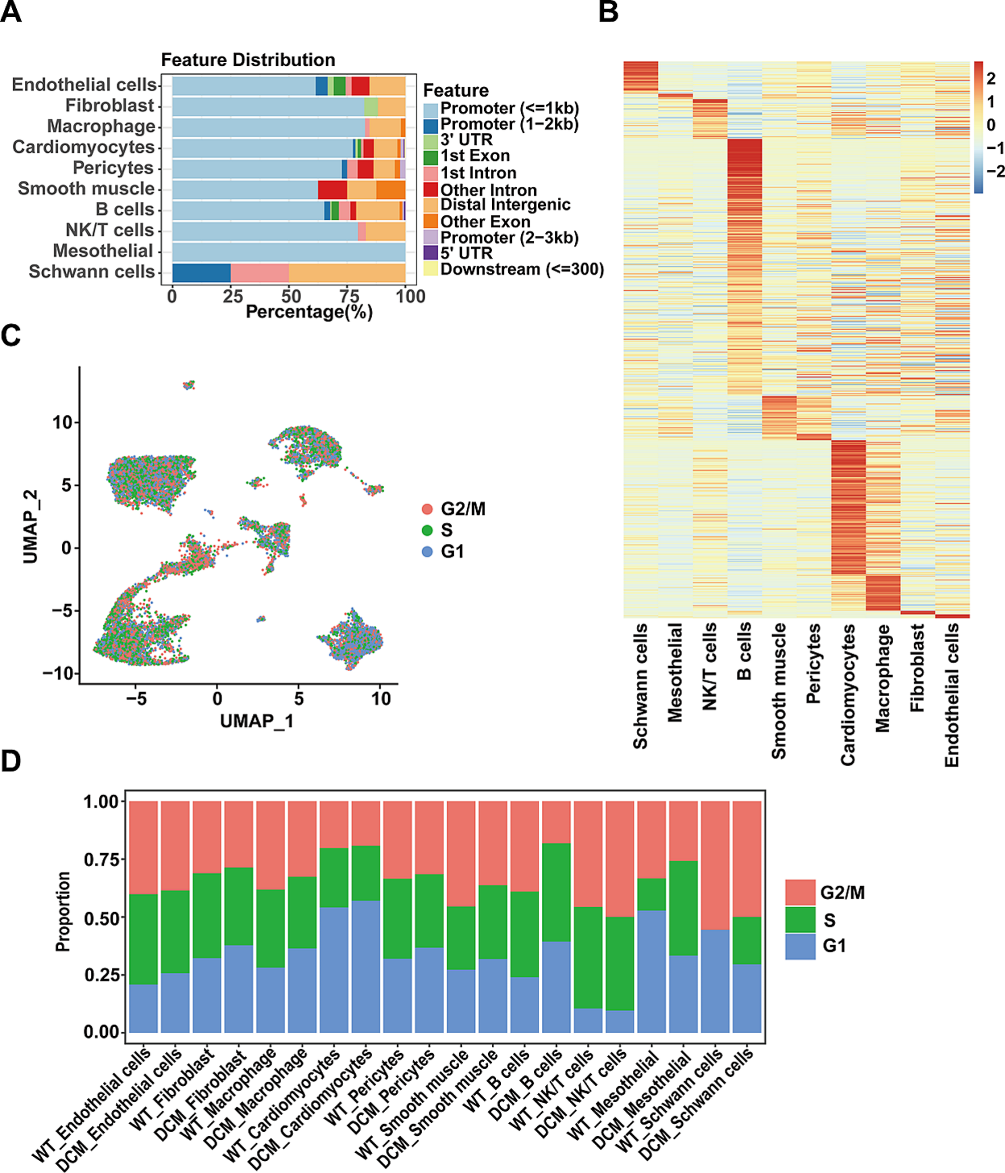
Distribution of cell type-specific chromatin accessible regions and cell cycle analysis. **A**. Bar plot of annotated DAR location annotation for each cell type. **B**. Heatmap of accessible level of differentially accessible region (DAR) for each cell type. The color scale represents the accessible level, which means the number of Tn5 sites within each DAR scaled by row. **C**. UMAP plot of multi-omics dataset and all cells are colored by the predicted classification (G2M, S, and G1 phase). **D**. Bar plot of cell phase for each cell type across all samples

The chromatin-accessible regions obtained from scATAC-seq can be categorized into the promoter, 1st exon, and its downstream regions. By comparing the proportions of different types of chromatin-accessible regions across various cell types, we observed that most cell types exhibit accessible regions in the promoter region. Notably, Schwann cells primarily harbor accessible regions in distal intergenic regions (Fig. 2A). Conducting differential analysis on the accessible regions revealed that myocardial cells possess the highest number of accessible regions, followed by B cells (Fig. 2B). This suggests heightened transcriptional regulatory activity in these two cell types. Further analysis of the cell cycle reveals that, compared to the WT group, the proportion of cells in the G1 phase is increased in all cells of the DCM group except for mesothelial and Schwann cells. This suggests a general decrease in proliferative capacity of parenchymal cells during DCM. Interestingly, in DCM, there is a significant increase in the proportion of mesothelial and Schwann cells in the S phase, while the proportion of cells entering the G2/M phase does not increase, indicating a potential cell cycle arrest (Fig. 2C and D).

### Subpopulation change of cardiomyocytes and fibroblasts during DCM

**Fig. 3:**
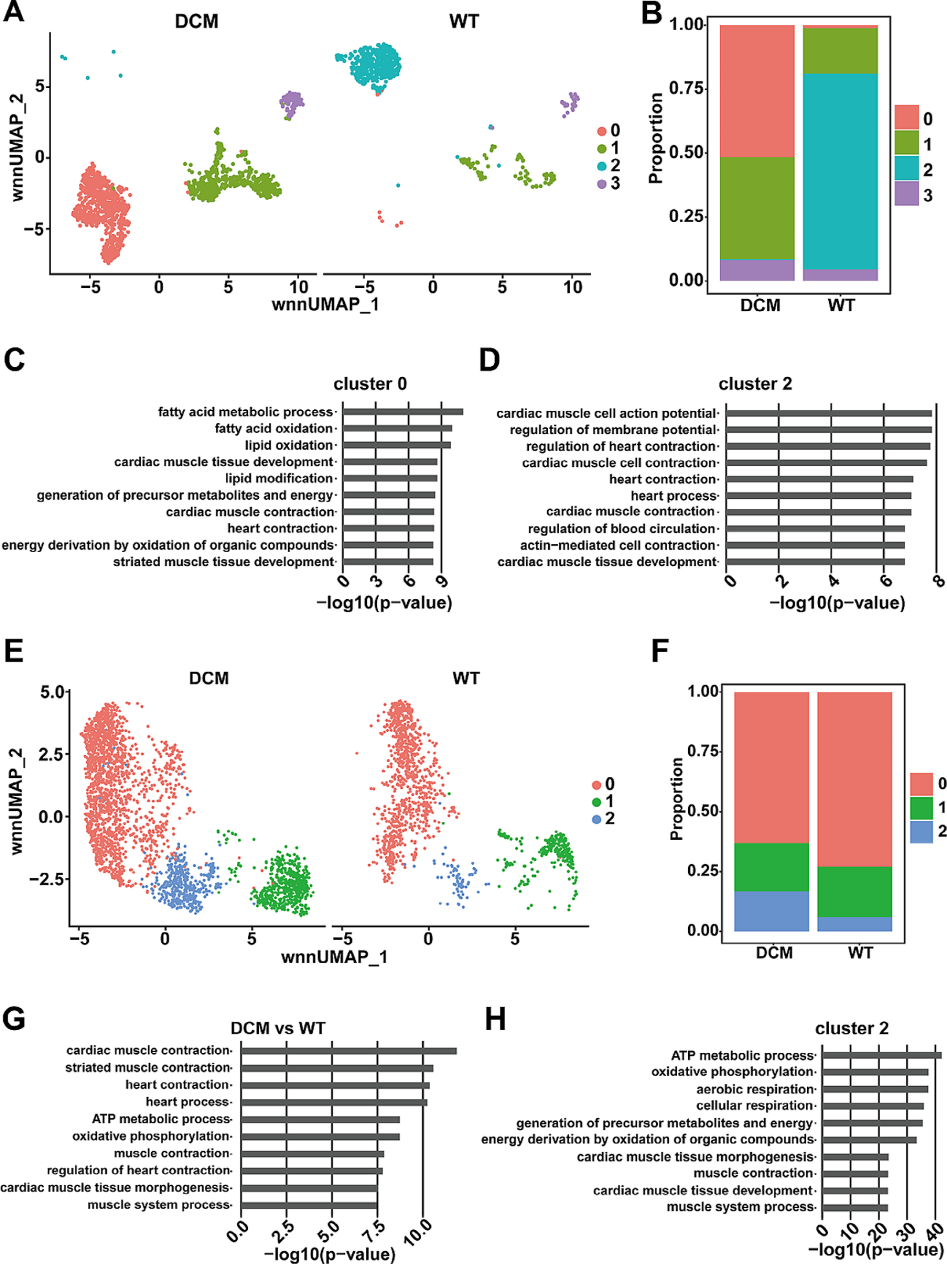
Subpopulation analysis of cardiomyocytes and fibroblasts. **A**. UMAP plot of cardiomyocytes re-clustering into four subpopulations. **B**. Bar plot of annotated subpopulations of cardiomyocytes for each sample. **C**. Bar plot of GO enrichment terms of differentially expressed genes in cardiomyocytes cluster 0. **D**. Bar plot of GO enrichment terms of differentially expressed genes in cluster 2 of cardiomyocytes. **E**. UMAP plot of fibroblast re-clustering into three subpopulations. **F**. Bar plot of annotated subpopulations of fibroblast for each sample. **G**. Bar plot of GO enrichment terms of upregulated genes in fibroblast of DCM versus WT groups. **H**. Bar plot of GO enrichment terms of differentially expressed genes in cluster 2 of fibroblast

Based on the previous analysis, we observed that myocardial cells and fibroblasts exhibit the most significant changes between the WT and DCM groups. We first focused on myocardial cells and performed additional dimensionality reduction and clustering, resulting in four subgroups: cardiomyocyte-0/1/2/3 (Fig. 3A and Figure S1A). Cell proportion comparison between the WT and DCM groups for each subgroup (Fig. 3B) revealed that cardiomyocyte-0 and cardiomyocyte-2 exhibited the most prominent changes. Specifically, cardiomyocyte-0 was predominantly distributed in the DCM group, while cardiomyocyte-2 showed an opposite distribution, being mostly present in the WT group. To further characterize these subgroups, we extracted specific marker genes for cardiomyocyte-0 and cardiomyocyte-2 and performed pathway enrichment analysis (Fig. 3C and D). The specific gene marker set for cardiomyocyte-0 was enriched in pathways related to fatty acid metabolism, fatty acid oxidation, and lipid oxidation, whereas the gene set for cardiomyocyte-2 was enriched in pathways related to myocardial action potentials, membrane potential regulation, and cardiac contraction, suggesting the damaged heart function during DCM.

Further dimensionality reduction and clustering of fibroblasts led to the identification of three subgroups: fibroblast-0/1/2 (Fig. 3E and Figure S1B). Cell proportion comparison between the WT and DCM groups for each fibroblast subgroup (Fig. 3F) indicated that fibroblast-0 and fibroblast-2 subgroups exhibited the most significant differences between these two groups. Specifically, fibroblast-0 showed a decrease in proportion in the DCM group, while the proportion of fibroblast-2 was markedly increased. Given the notable increase in the proportion of fibroblasts in the DCM group, we first performed differential analysis on all fibroblasts between the WT and DCM groups. The resulting set of differentially expressed genes was functionally enriched, revealing an enrichment in contraction-related pathways (Fig. 3G). Furthermore, we extracted the specific gene set for the fibroblast-2 subgroup, which exhibited a significantly increased proportion in the DCM group, and performed pathway enrichment analysis. The specific gene marker set for fibroblast-2 was found to be primarily enriched in energy metabolism and contraction pathways (Fig. 3H).

### Subpopulation analysis of endothelial cell

**Fig. 4:**
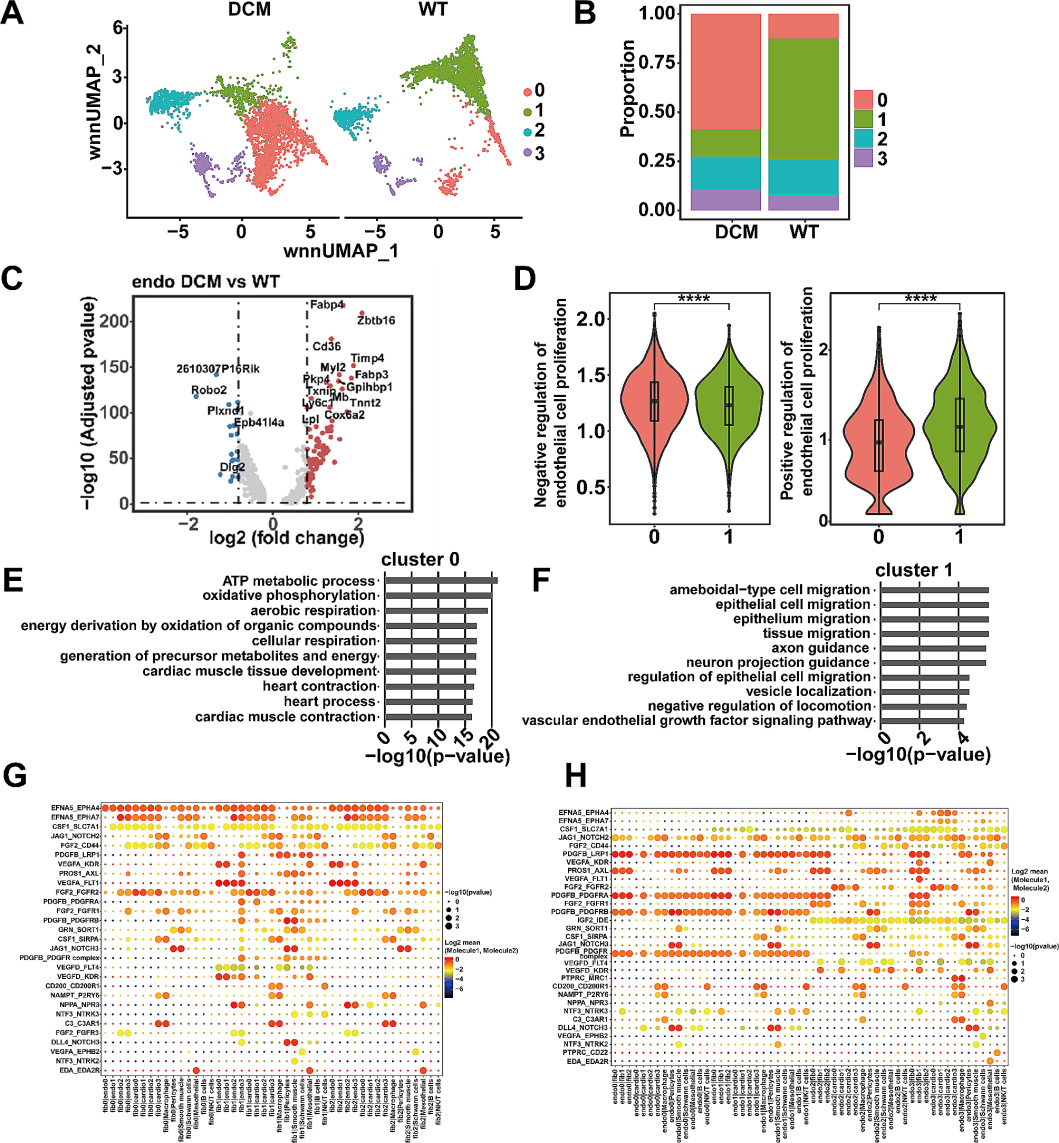
Subpopulation analysis of endothelial cells. **A**. UMAP plot of endothelial cells re-clustering into four subpopulations. **B**. Bar plot of annotated subpopulations of endothelial cells for each sample. **C**. Volcano plot of the fold-change in differential gene expression in WT and DCM groups. Wilcoxon rank-sum test was used to calculate *P*-values and the Bonferroni analysis was used to calculate adjusted *P* values. **D**. Violin plot of biological processes scores defined as average z-scores of process-related genes (right) for selected subpopulations. Significance was determined by the Wilcoxon test and divided into three levels (NS, not significant (*P* > 0.05); *** *P* < 0.001; **** *P* < 0.0001). **E**. Bar plot of GO enrichment terms of differentially expressed genes in cluster 0 of endothelial cells. **F**. Bar plot of GO enrichment terms of differentially expressed genes in cluster 1 of endothelial cells. **G**. Overview of ligand-receptor interactions between fibroblast and other cell types; circle size represents *P*-values, scale on the right. The color scale represents the means of the average expression level of ligands and receptors in the corresponding cluster. **H**. Overview of ligand-receptor interactions between endothelial cells and other cell types; circle size represents *P*-values, scale on the right. The color scale represents the means of the average expression level of ligands and receptors in the corresponding cluster

In response to the decreased proportion of endothelial cells in the DCM group, we similarly extracted and performed dimensionality reduction and clustering to obtain four subgroups: endothelial cell-0/1/2/3 (Fig. 4A). Further cell proportion comparison between the WT and DCM groups for each endothelial cell subgroup (Fig. 4B) highlighted endothelial cell-0 and endothelial cell-1 as exhibiting the most notable changes between these two groups. Specifically, endothelial cell-0 was predominantly distributed in the DCM group, while endothelial cell-1 was mainly present in the WT group. Differential analysis between endothelial cells in the two groups revealed genes such as Fabp4, Zbtb16, and Cd36 being upregulated in the DCM group, while Robo2, Plxnd1, and Dlg2 were highly expressed in the WT group (Fig. 4C). Corresponding to the decrease in endothelial cells in the DCM group, genes specifically expressed in endothelial cell-0 were associated with inhibiting endothelial cell proliferation, while those in endothelial cell-1 were associated with promoting endothelial cell proliferation (Fig. 4D). Pathway enrichment analysis of the gene sets specifically expressed in endothelial cell-0 and endothelial cell-1 subgroups (Fig. 4E and F) indicated that genes specifically expressed in endothelial cell-0 significantly enriched in contraction-related pathways, including heart contraction. Interestingly, this result is consistent with the pathway enrichment observed in the differential gene analysis of fibroblasts. On the other hand, genes specifically expressed in endothelial cell-1 significantly enriched in growth-related pathways, such as the VEGF signaling pathway.

### Analysis of cell communication centered on fibroblasts and endothelial cells

To explore the interaction of fibroblasts and endothelial cells with other cells, we conducted cell-cell communication analysis between cell subgroups, focusing on ligand expression by fibroblasts (Fig. 4G) and endothelial cell (Fig. 4H) subgroups regulating other cells. We identified interactions between fibroblast-1 and fibroblast-2 subgroups with endothelial cells involving receptors such as VEGFA-FLT1 and VEGFA-KDR, which include VEGF receptors. Previous research has suggested their relevance to heart diseases [26]. For example, VEGF-A is key in triggering cardiac angiogenic responses after acute myocardial infarction via regulating its interactors or downstream factors [27, 28]. The results indicated that both endothelial cell-0 and endothelial cell-1 exhibited interactions with myocardial cells and fibroblasts involving receptors such as PDGFB_LRP1, PROS1_AXL, PDGFB_PDGFRA, PDGFB_PDGFRB, and PDGFB_PDGFR (Fig. 4H).

### Candidate regulators in fibroblast activation and endothelial proliferation

**Fig. 5:**
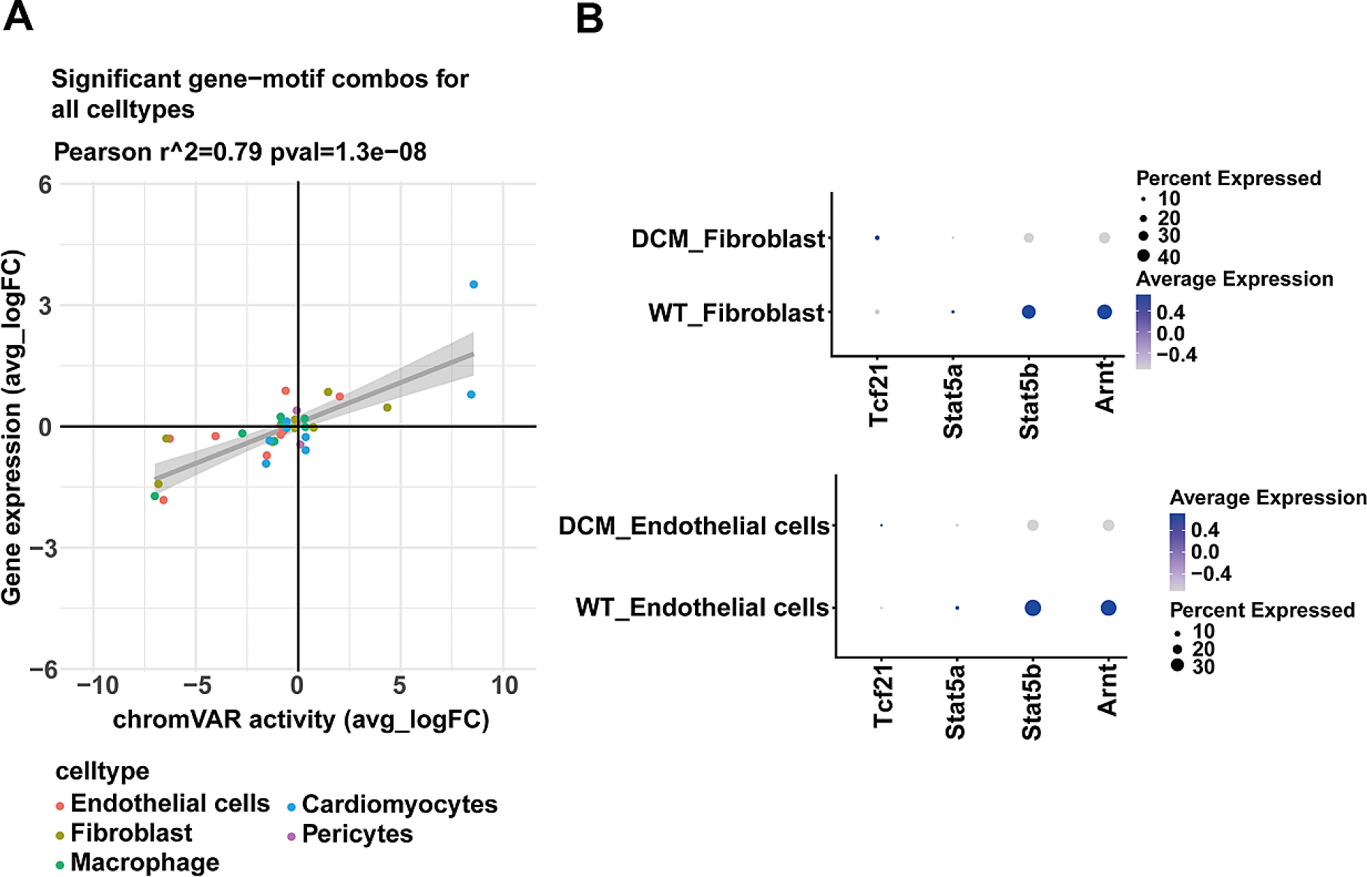
Analysis of potential transcription factors. **A**. The mean values of motif activity calculated by chromVAR in each cell type were correlated with the mean values of gene expression levels of transcription factors from the JASPAR database. **B**. Expression of the transcription factors Arnt, Stat5a, and Stat5b in Fibroblasts and Endothelial Cells between DCM and WT Groups. The diameter of the dot represents the proportion of cells that express the corresponding gene and the density of the dot represents the average gene expression level among all cell types

To explore key regulators in different cell types, we conducted a correlation analysis between the mean motif activity calculated through chromVAR and the mean gene expression levels of transcription factors from the JASPAR database in each cell type (Fig. 5A). The results indicated a significant positive correlation between motif activity and the expression of transcription factor-encoding genes in endothelial cells, fibroblasts, macrophages, myocardial cells, and peripheral cells. Subsequently, we identified 23 transcription factors positively correlated with motif activity, including Creb5, Esrrg, Sox17, Esrra, Tcf21, Ddit3, Nr1h3, and others. Additionally, we identified 13 transcription factors negatively correlated with motif activity, such as Ahr, Rxra, Arnt, Stat5a, and Stat5b.

Through exploration of the transcription factors, we identified Tcf21, Arnt, Stat5a, and Stat5b as potentially key players in the development of diabetic cardiomyopathy, particularly in regulating endothelial cells and myocardial cells. Acharya et al. previously proposed that the loss of Tcf21 prevents the formation of cardiac fibroblasts, indicating the importance of this transcription factor in determining fibroblast fate [29]. Correspondingly, our results also revealed an upregulation of Tcf21 in the DCM group (Fig. 5B). Existing research suggests that a decrease in Arnt can lead to vascular dysfunction in diabetic patients [30], while Stat5 positively regulates vascular angiogenesis [31]. This suggests that Arnt, Stat5a, and Stat5b may positively regulate endothelial cell proliferation. This is consistent with the reduced expression of Arnt, Stat5a, and Stat5b observed in the DCM group in our study.

### Markers for cell subpopulations during DCM

**Fig. 6:**
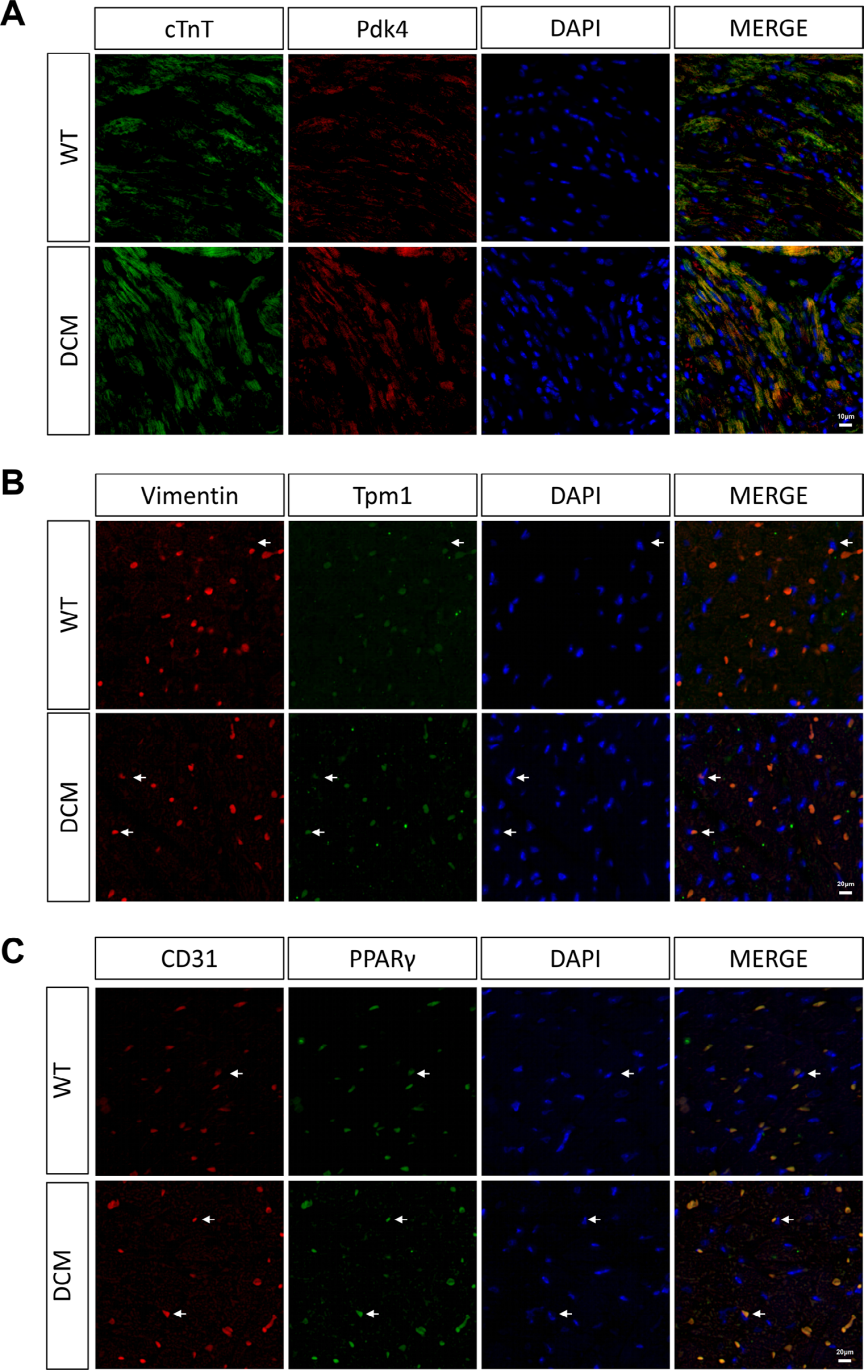
Comparison of marker protein in fibroblasts between DCM and WT groups. **A**. Expression of Pdk4 protein in fibroblasts from DCM and WT groups is shown by immunofluorescence staining, where blue is the DAPI dye, green is the cTnT protein, and red is the Pdk4 protein. **B**. Expression of Tpm1 protein in fibroblasts from DCM and WT groups is shown by immunofluorescence staining, where blue is the DAPI dye, green is the Tpm1 protein, and red is the Vimentin protein. **C**. Expression of PPARγ protein in fibroblasts from DCM and WT groups is shown by immunofluorescence staining, where blue is the DAPI dye, green is the PPARγ protein, and red is the CD31 protein

To validate the marker genes inferred by scRNA-seq data in cardiomyocytes, fibroblast and endothelial cells, we performed fluorescence staining for the Pdk4 protein in cardiomyocytes (inferred marker for cardiomyocyte cluster 0), Tpm1 protein in fibroblasts (inferred marker for fibroblast cluster 2) and the PPARγ protein (inferred marker for endothelial cell cluster 0) in endothelial cells (Fig. 6A-C). The expression of the Pdk4 protein was higher in cardiomyocytes of the DCM group compared to the WT group. And the expression of the Tpm1 protein was higher in fibroblasts of the DCM group compared to the WT group. Moreover, the expression of the PPARγ protein was higher in endothelial cells of the DCM group compared to the WT group. These results consistently confirmed that Pdk4, PPARγ, and Tpm1 as markers of cardiomyocytes with altered metabolic patterns, endothelial cells with compromised proliferation, and activated fibroblasts, respectively. Furthermore, the mining of public datasets GSE131779 indicated the presence of the binding sites of transcription factor TCF21 on TPM1 and PPARγ gene [32], further suggesting the key roles of the Tcf21 regulatory network in DCM development.

## Discussion

Heart failure and cardiovascular disease are the leading causes of death in diabetic patients. The pathogenesis of Diabetic Cardiomyopathy (DCM) involves various cellular pathophysiological processes of cardiomyocytes, fibroblasts, endothelial cells, etc. Understanding the molecular mechanisms of diabetic cardiomyopathy and heart failure, and finding new therapeutic strategies, are some of the major challenges in cardiovascular endocrinology [33, 34]. In this study, we investigated the cellular changes, chromatin accessibility, and characteristics of relevant cell subpopulations during the development of diabetic cardiomyopathy (DCM) by integrating single-cell RNA sequencing (scRNA-seq) and single-cell transposase-accessible chromatin sequencing (scATAC-seq) data. Our clustering analysis of the integrated data was robust and accurate, revealing significant alterations in cell proportions and functional states. Specifically, in a DCM mouse model, we observed a decrease in the proportion of endothelial cells and macrophages, accompanied by an increase in fibroblasts and cardiomyocytes, implying exacerbation of cardiac fibrosis and endothelial damage. Additionally, enhanced transcriptional regulatory activity was observed in cardiomyocytes, while the proliferative capacity of substantial cells was universally reduced. Furthermore, heterogeneity within cardiomyocytes, fibroblasts, and endothelial cells was identified, with each subpopulation displaying distinct functional characteristics. Analysis of intercellular communication revealed interactions between fibroblasts, endothelial cells, and other cardiac cell types. Additionally, potential regulatory factors such as Tcf21, Arnt, Stat5a, and Stat5b, were identified, suggesting their pivotal roles in the pathogenesis of DCM. Immunofluorescence staining validated the accuracy of marker genes for metabolic reprogramming in cardiomyocytes, activated fibroblasts, and proliferative-impaired endothelial cells. In summary, our study provides a detailed description of the complex cellular states and molecular changes in DCM, offering valuable insights into potential therapeutic targets.

DCM is characterized by adverse structural remodeling, including cardiac hypertrophy and fibrosis, as well as early diastolic and late systolic dysfunction [35]. Identifying subclinical left ventricular diastolic dysfunction (LVDD) holds significant clinical importance for its prevention. Novel methods such as the myocardial performance index (MPI) and Presystolic wave (PSW) measurement improve the assessment of subclinical LVDD [36]. Dysregulation of cardiomyocytes, fibroblasts, endothelial cells, and immune cells in DCM promotes pathological cardiac remodeling, ultimately leading to heart failure in diabetic patients. Consistent with previous studies [24, 25], we observed an increase in the proportion of cardiomyocytes and fibroblasts, along with a decrease in endothelial cells and immune cells in DCM, indicating exacerbation of fibrosis and endothelial injury during disease progression. Subsequent subpopulation analysis revealed metabolic alterations and impaired contractile function-related subpopulations within cardiomyocytes, reflecting the detrimental effects of DCM on the myocardium. Abnormal glucose metabolism in early diabetes leads to increased fatty acid β-oxidation to compensate for insufficient energy in the diabetic heart [37]. Numerous studies using transgenic animal models have shown that upregulation of myocardial fatty acid transport proteins leads to increased myocardial fatty acid uptake and lipotoxicity, exacerbating DCM development [35, 38]. Dysregulated glucose and lipid metabolism lead to abnormal regulation of cell signaling pathways related to inflammation and oxidative stress, contributing to the development of cardiac hypertrophy, fibrosis, and heart failure in diabetes [24, 39, 40]. Although first-line drugs targeting glucose/lipid metabolism such as metformin, thiazolidinediones, and sodium-glucose cotransporter-2 inhibitors alleviate hyperglycemia, their exact effects on DCM need further exploration in diabetic animal models and patients. A recent clinical study showed that metformin treatment reduces the risk of death in critically ill patients with type 2 diabetes mellitus and chronic heart failure [41].

Similarly, subpopulations of activated fibroblasts in DCM displayed differences in energy metabolism and pathways related to contraction, suggesting their crucial role in the development of DCM. These differences may reflect functional and epigenetic changes in fibroblasts during cardiac remodeling and fibrosis. Previous studies have indicated that myocardial fibrosis is a significant pathological process in DCM, closely associated with impaired cardiac function [42]. Autopsy of diabetic patients shows collagen accumulation in the heart, manifested as perivascular, interstitial, or replacement fibrosis, indicating that cardiac fibrosis is a major cause of heart failure in DCM [43, 44]. The transition of cardiac fibroblasts (CFs) to myofibroblasts is a core cellular event in cardiac fibrosis in DCM, involving multiple cell types, including cardiomyocytes, endothelial cells, and immune cells, responding to oxidative stress, endoplasmic reticulum stress, and inflammation, attributable to sustained metabolic disturbances in DCM [45]. Myofibroblasts are contractile and secreting cell types involved in cardiac fibrosis remodeling by producing extracellular matrix (ECM) proteins [46]. The fibroblast cluster 2 that we identified is enriched in pathways related to contractile function. Previous studies have found that Fibroblasts isolated from the hearts of diabetic patients show enhanced proliferation activity and high collagen expression [47]. Additionally, in vitro experiments have shown that high glucose (HG) treatment promotes proliferation and collagen formation in CFs [48, 49]. Furthermore, consistent with our findings, in DCM, insulin resistance leads to a shift in myocardial cells towards lipid metabolism, which increased metabolic pressure and oxidative stress lead to myocardial cell damage [50], releasing DAMP proteins that activate CFs to promote myocardial fibrosis. Moreover, Immune cells, monocytes/macrophages (Mo/Mf), not only indirectly affect cardiac fibrosis through pro-inflammatory cytokines but also directly differentiate into myofibroblasts under the action of various cytokines [51]. Endothelial cells can also undergo endothelial-to-mesenchymal transition (EndMT) and further differentiate into myofibroblasts [7–9]. Moreover, fibrotic mediators produced by endothelial cells also participate in the proliferation and differentiation of cardiac myofibroblasts [52, 53]. In our study, we observed an increase in the proportion of fibroblasts and a decrease in the proportions of monocytes and endothelial cells in DCM. Further intercellular communication analysis revealed cross-talk between proliferatively impaired endothelial cells and activated fibroblasts involving factors such as PDGFB/PDGFR and VEGFA/FLT1. PDGF [54] and VEGF are well-known mediators of fibrosis and angiogenesis, and inhibiting PDGF, VEGF, and FGF signaling pathways can attenuate fibrosis [55]. These findings suggest that fibroblasts and endothelial cells play crucial roles in the development and progression of DCM.

In this study, we investigated the chromatin accessibility in DCM to explore the activity status of specific genomic regions and revealed its close correlation with gene transcriptome expression. It is also important to note the role of epigenetics, which refers to the phenomenon of regulating gene expression through DNA methylation, histone modifications, and other mechanisms without involving changes in DNA sequence in the genome. Previous research has shown association between epigenetic changes and the development of DCM, particularly involving processes mediated by histone deacetylases (HDACs), HDACs could regulate cardiovascular and metabolic diseases in cellular processes including cardiac fibrosis, hypertrophy, oxidative stress and inflammation [56]. SIRT1, a Class III HDAC, may exert a protective effect on DCM through histone deacetylation [57]. Based on scRNA-seq and scATAC-seq data, we also explored key regulatory factors in different cell types, identifying Tcf21, Arnt, Stat5a, and Stat5b as potentially playing crucial roles in the occurrence and development of DCM, particularly in regulating fibroblasts and endothelial cells. Previous studies have shown that the loss of Tcf21 can prevent CF formation, highlighting its importance in determining fibroblast fate [29]. We observed upregulation of Tcf21 in DCM, suggesting its potential as a key regulatory factor in cardiac fibrosis in DCM. Furthermore, decreased expression of Arnt has been associated with vascular dysfunction in diabetic patients [30], while Stat5 has been found to positively regulate angiogenesis [31]. We observed decreased expression of Arnt, Stat5a, and Stat5b in the DCM group, further supporting their potential roles in regulating endothelial cell proliferation. While the pathogenesis of diabetic cardiomyopathy remains unclear, potential mechanisms include insulin resistance, alterations in substrate metabolism, mitochondrial dysfunction, increased oxidative stress, secretion of adipokines, disrupted signaling pathways, and impaired calcium homeostasis [58]. Our findings provide important clues for further understanding the pathogenesis of diabetic cardiomyopathy. In addition to Tcf21, Arnt, Stat5a, and Stat5b, previous studies have explored and demonstrated the significant roles of other transcription factors in DCM. For instance, ATF4 [59] in DCM promotes cardiac fibrosis and oxidative stress, while downregulation of Bmal1 [60] induces mitochondrial dysfunction by promoting Bcl2/IP3R-mediated mitochondrial Ca^2+^ overload, leading to diabetic cardiomyopathy. A recent study has also revealed that ketone bodies can rescue mitochondrial dysfunction through epigenetic remodeling [61]. The role of calcium homeostasis in DCM is multifaceted, as evidenced by a study investigating the involvement of Ca^2+^ in adipokine resistin-induced activation. Adipokine resistin, believed to be associated with obesity, insulin resistance, and diabetes, is highly expressed in DCM [58].

Finally, we identified Pdk4, Tpm1, and PPARγ as markers for metabolic reprogramming in cardiomyocytes, activated fibroblasts, and proliferative impaired endothelial cell subpopulations identified by scRNA-seq in DCM mice, and further confirmed these findings through fluorescence staining. Interestingly, in previous study, TCF21 binding sites was observed on TPM1 and PPARγ genes [32], further emphasizing the potential key role of the TCF21 regulatory network in DCM. Moreover, a recent study has demonstrated that USP28 can directly interact with PPARα (Lys152) and exert a protective effect in DCM by regulating mitochondrial homeostasis through the PPARα-Mfn2 axis.

In conclusion, we have revealed subpopulations of metabolic reprogramming cardiomyocytes, activated fibroblasts with contractile functions, and proliferative impaired endothelial cells in DCM, discussed the functional roles of these three cell subpopulations in the occurrence and development of DCM, explored their potential interactions, and discussed potential key regulatory factors. We have validated their existence in DCM, providing potential candidate drugs and targets for the diagnosis and treatment of diabetic cardiomyopathy, opening up new avenues and directions for the development of novel drugs and intervention strategies. While integrating the chromatin accessibility information provided by ATAC-seq with the transcriptome information provided by scRNA-seq allows us to gain a more comprehensive understanding of the functional state and characteristics of cells in DCM, to more accurately identify and characterize cell types, to dissect the roles of different cell subpopulations in DCM development, and to reveal the dynamic changes in gene expression and chromatin accessibility of cells in DCM, providing new perspectives and methods for understanding the development process of DCM. However, there are also some limitations. The data analysis and integration of ATAC-seq and scRNA-seq results may be influenced by the analysis methods. Additionally, despite our good sequencing quality assessment, there may still be some biases in the experimental process, such as PCR amplification bias and uneven sequencing depth, which could affect the accuracy and reliability of the data. Furthermore, our study only used one type of diabetic mouse model, which may not fully capture the complexity and heterogeneity of human diabetic cardiomyopathy. Future studies may consider using different diabetic mouse models or human samples to validate and extend the results, enhancing the robustness and applicability of the findings. Although we identified some potential regulatory networks, their specific mechanisms need further validation and exploration.

### Electronic supplementary material

Below is the link to the electronic supplementary material.

Supplementary Material 1

Supplementary Material 2

Supplementary Material 3

Supplementary Material 4

## Data Availability

Sequence data that support the findings of this study have been deposited in the Sequence Read Archive with the primary accession code PRJNA1069235, and others data were provided within the manuscript or supplementary information files.

## References

[1] Gollmer J, Zirlik A, Bugger H (2019). Established and emerging mechanisms of Diabetic Cardiomyopathy. J Lipid Atheroscler.

[2] Sun H, Saeedi P, Karuranga S, Pinkepank M, Ogurtsova K, Duncan BB, Stein C, Basit A, Chan JCN, Mbanya JC (2022). IDF Diabetes Atlas: Global, regional and country-level diabetes prevalence estimates for 2021 and projections for 2045. Diabetes Res Clin Pract.

[3] Sacre JW, Magliano DJ, Shaw JE (2021). Heart failure hospitalisation relative to major atherosclerotic events in type 2 diabetes with versus without chronic kidney disease: a meta-analysis of cardiovascular outcomes trials. Diabetes Metab.

[4] Palazzuoli A, Iacoviello M (2023). Diabetes leading to heart failure and heart failure leading to diabetes: epidemiological and clinical evidence. Heart Fail Rev.

[5] Yang J, Liu Y, Fan X, Li Z, Cheng Y (2014). A pathway and network review on beta-adrenoceptor signaling and beta blockers in cardiac remodeling. Heart Fail Rev.

[6] Tuleta I, Frangogiannis NG (2021). Fibrosis of the diabetic heart: clinical significance, molecular mechanisms, and therapeutic opportunities. Adv Drug Deliv Rev.

[7] Piera-Velazquez S, Jimenez SA (2019). Endothelial to mesenchymal transition: role in physiology and in the Pathogenesis of Human diseases. Physiol Rev.

[8] Nakamura K, Miyoshi T, Yoshida M, Akagi S, Saito Y, Ejiri K, Matsuo N, Ichikawa K, Iwasaki K, Naito T et al. Pathophysiology and Treatment of Diabetic Cardiomyopathy and Heart failure in patients with diabetes Mellitus. Int J Mol Sci 2022, 23(7). 10.3390/ijms23073587 PMC8999085 35408946

[9] Du JK, Yu Q, Liu YJ, Du SF, Huang LY, Xu DH, Ni X, Zhu XY (2021). A novel role of kallikrein-related peptidase 8 in the pathogenesis of diabetic cardiac fibrosis. Theranostics.

[10] Phang RJ, Ritchie RH, Hausenloy DJ, Lees JG, Lim SY (2023). Cellular interplay between cardiomyocytes and non-myocytes in diabetic cardiomyopathy. Cardiovasc Res.

[11] Li W, Lou X, Zha Y, Qin Y, Zha J, Hong L, Xie Z, Yang S, Wang C, An J et al. Single-cell RNA-seq of heart reveals intercellular communication drivers of myocardial fibrosis in diabetic cardiomyopathy. Elife 2023, 12. 10.7554/eLife.80479 PMC10110238 37010266

[12] Sinha S, Satpathy AT, Zhou W, Ji H, Stratton JA, Jaffer A, Bahlis N, Morrissy S, Biernaskie JA (2021). Profiling chromatin accessibility at single-cell resolution. Genomics Proteom Bioinf.

[13] Butler A, Hoffman P, Smibert P, Papalexi E, Satija R (2018). Integrating single-cell transcriptomic data across different conditions, technologies, and species. Nat Biotechnol.

[14] Stuart T, Srivastava A, Madad S, Lareau CA, Satija R (2021). Single-cell chromatin state analysis with Signac. Nat Methods.

[15] McGinnis CS, Murrow LM, Gartner ZJ (2019). DoubletFinder: Doublet Detection in single-cell RNA sequencing data using Artificial Nearest neighbors. Cell Syst.

[16] Gaspar JM. Improved peak-calling with MACS2. *BioRxiv* 2018:496521.

[17] Hao Y, Hao S, Andersen-Nissen E, Mauck WM, Zheng S, Butler A, Lee MJ, Wilk AJ, Darby C, Zager M (2021). Integrated analysis of multimodal single-cell data. Cell.

[18] Schep AN, Wu B, Buenrostro JD, Greenleaf WJ (2017). chromVAR: inferring transcription-factor-associated accessibility from single-cell epigenomic data. Nat Methods.

[19] Pliner HA, Packer JS, McFaline-Figueroa JL, Cusanovich DA, Daza RM, Aghamirzaie D, Srivatsan S, Qiu X, Jackson D, Minkina A (2018). Cicero predicts cis-Regulatory DNA interactions from single-cell chromatin Accessibility Data. Mol Cell.

[20] Fishilevich S, Nudel R, Rappaport N, Hadar R, Plaschkes I, Iny Stein T, Rosen N, Kohn A, Twik M, Safran M et al. GeneHancer: genome-wide integration of enhancers and target genes in GeneCards. *Database (Oxford)* 2017, 2017. 10.1093/database/bax028 PMC5467550 28605766

[21] Yu G, Wang LG, Han Y, He QY (2012). clusterProfiler: an R package for comparing biological themes among gene clusters. OMICS.

[22] The Gene Ontology C (2019). The Gene Ontology Resource: 20 years and still GOing strong. Nucleic Acids Res.

[23] Vento-Tormo R, Efremova M, Botting RA, Turco MY, Vento-Tormo M, Meyer KB, Park JE, Stephenson E, Polanski K, Goncalves A (2018). Single-cell reconstruction of the early maternal-fetal interface in humans. Nature.

[24] Jia G, Hill MA, Sowers JR (2018). Diabetic Cardiomyopathy: an update of mechanisms contributing to this clinical entity. Circ Res.

[25] Wang M, Li Y, Li S, Lv J (2022). Endothelial dysfunction and Diabetic Cardiomyopathy. Front Endocrinol (Lausanne).

[26] Braile M, Marcella S, Cristinziano L, Galdiero MR, Modestino L, Ferrara AL, Varricchi G, Marone G, Loffredo S. VEGF-A in Cardiomyocytes and Heart diseases. Int J Mol Sci 2020, 21(15). 10.3390/ijms21155294 PMC7432634 32722551

[27] Gao L, Yang J, Li Y, Liu K, Sun H, Tang J, Xia Z, Zhang L, Hu Z. Long Noncoding RNA SCIRT Promotes HUVEC Angiogenesis via Stabilizing VEGFA mRNA Induced by Hypoxia. *Oxid Med Cell Longev* 2022, 2022:9102978. 10.1155/2022/9102978 PMC9187973 35698607

[28] Zhao T, Zhao W, Chen Y, Ahokas RA, Sun Y (2010). Vascular endothelial growth factor (VEGF)-A: role on cardiac angiogenesis following myocardial infarction. Microvasc Res.

[29] Acharya A, Baek ST, Huang G, Eskiocak B, Goetsch S, Sung CY, Banfi S, Sauer MF, Olsen GS, Duffield JS (2012). The bHLH transcription factor Tcf21 is required for lineage-specific EMT of cardiac fibroblast progenitors. Development.

[30] Knapp M, Zheng M, Sladojevic N, Zhao Q, Liao JK, Wu R (2016). Reduction of endothelial Arnt mediates vascular dysfunction in diabetes. Circulation.

[31] Yang X, Meyer K, Friedl A (2013). STAT5 and prolactin participate in a positive autocrine feedback loop that promotes angiogenesis. J Biol Chem.

[32] Wirka RC, Wagh D, Paik DT, Pjanic M, Nguyen T, Miller CL, Kundu R, Nagao M, Coller J, Koyano TK (2019). Atheroprotective roles of smooth muscle cell phenotypic modulation and the TCF21 disease gene as revealed by single-cell analysis. Nat Med.

[33] Jankauskas SS, Kansakar U, Varzideh F, Wilson S, Mone P, Lombardi A, Gambardella J, Santulli G (2021). Heart failure in diabetes. Metabolism.

[34] Varzideh F, Kansakar U, Jankauskas SS, Gambardella J, Santulli G (2021). Cardiovascular Endocrinology: evolving concepts and updated epidemiology of relevant diseases. Front Endocrinol (Lausanne).

[35] Tan Y, Zhang Z, Zheng C, Wintergerst KA, Keller BB, Cai L (2020). Mechanisms of diabetic cardiomyopathy and potential therapeutic strategies: preclinical and clinical evidence. Nat Rev Cardiol.

[36] Askin L, Tanrıverdi O, Tibilli H, Turkmen S (2019). New Method improves the evaluation of subclinical left ventricular dysfunction in type 2 diabetes Mellitus. Arq Bras Cardiol.

[37] Kim JA, Wei Y, Sowers JR (2008). Role of mitochondrial dysfunction in insulin resistance. Circ Res.

[38] Chiu HC, Kovacs A, Blanton RM, Han X, Courtois M, Weinheimer CJ, Yamada KA, Brunet S, Xu H, Nerbonne JM (2005). Transgenic expression of fatty acid transport protein 1 in the heart causes lipotoxic cardiomyopathy. Circ Res.

[39] Jia G, Whaley-Connell A, Sowers JR (2018). Diabetic cardiomyopathy: a hyperglycaemia- and insulin-resistance-induced heart disease. Diabetologia.

[40] Chen MY, Meng XF, Han YP, Yan JL, Xiao C, Qian LB (2022). Profile of crosstalk between glucose and lipid metabolic disturbance and diabetic cardiomyopathy: inflammation and oxidative stress. Front Endocrinol (Lausanne).

[41] Guo Q, Hong W, Chen J, Zhu X, Duan G, Huang H, Duan C. Metformin Treatment Is Associated with Mortality in patients with type 2 diabetes and chronic heart failure in the Intensive Care Unit: a retrospective cohort study. Cardiovasc Innovations Appl 2023, 8(1).

[42] Asbun J, Villarreal FJ (2006). The pathogenesis of myocardial fibrosis in the setting of Diabetic Cardiomyopathy. J Am Coll Cardiol.

[43] Rubler S, Dlugash J, Yuceoglu YZ, Kumral T, Branwood AW, Grishman A (1972). New type of cardiomyopathy associated with diabetic glomerulosclerosis. Am J Cardiol.

[44] Regan TJ, Lyons MM, Ahmed SS, Levinson GE, Oldewurtel HA, Ahmad MR, Haider B (1977). Evidence for cardiomyopathy in familial diabetes mellitus. J Clin Invest.

[45] Cheng Y, Wang Y, Yin R, Xu Y, Zhang L, Zhang Y, Yang L, Zhao D (2023). Central role of cardiac fibroblasts in myocardial fibrosis of diabetic cardiomyopathy. Front Endocrinol (Lausanne).

[46] Souders CA, Bowers SL, Baudino TA (2009). Cardiac fibroblast: the Renaissance cell. Circ Res.

[47] Fowlkes V, Clark J, Fix C, Law BA, Morales MO, Qiao X, Ako-Asare K, Goldsmith JG, Carver W, Murray DB (2013). Type II diabetes promotes a myofibroblast phenotype in cardiac fibroblasts. Life Sci.

[48] Yang XX, Zhao ZY (2022). miR-30a-5p inhibits the proliferation and collagen formation of cardiac fibroblasts in diabetic cardiomyopathy. Can J Physiol Pharmacol.

[49] Zhao T, Chen H, Cheng C, Zhang J, Yan Z, Kuang J, Kong F, Li C, Lu Q (2019). Liraglutide protects high-glucose-stimulated fibroblasts by activating the CD36-JNK-AP1 pathway to downregulate P4HA1. Biomed Pharmacother.

[50] Jia G, DeMarco VG, Sowers JR (2016). Insulin resistance and hyperinsulinaemia in diabetic cardiomyopathy. Nat Rev Endocrinol.

[51] Cojan-Minzat BO, Zlibut A, Agoston-Coldea L (2021). Non-ischemic dilated cardiomyopathy and cardiac fibrosis. Heart Fail Rev.

[52] Adiarto S, Heiden S, Vignon-Zellweger N, Nakayama K, Yagi K, Yanagisawa M, Emoto N (2012). ET-1 from endothelial cells is required for complete angiotensin II-induced cardiac fibrosis and hypertrophy. Life Sci.

[53] Widyantoro B, Emoto N, Nakayama K, Anggrahini DW, Adiarto S, Iwasa N, Yagi K, Miyagawa K, Rikitake Y, Suzuki T (2010). Endothelial cell-derived endothelin-1 promotes cardiac fibrosis in diabetic hearts through stimulation of endothelial-to-mesenchymal transition. Circulation.

[54] Klinkhammer BM, Floege J, Boor P (2018). PDGF in organ fibrosis. Mol Aspects Med.

[55] Chaudhary NI, Roth GJ, Hilberg F, Müller-Quernheim J, Prasse A, Zissel G, Schnapp A, Park JE (2007). Inhibition of PDGF, VEGF and FGF signalling attenuates fibrosis. Eur Respir J.

[56] Ke X, Lin Z, Ye Z, Leng M, Chen B, Jiang C, Jiang X, Li G (2021). Histone Deacetylases in the Pathogenesis of Diabetic Cardiomyopathy. Front Endocrinol (Lausanne).

[57] Wang AJ, Wang S, Wang BJ, Xiao M, Guo Y, Tang Y, Zhang J, Gu J (2020). Epigenetic regulation Associated with Sirtuin 1 in complications of diabetes Mellitus. Front Endocrinol (Lausanne).

[58] Singh R, Moreno P, Hajjar RJ, Lebeche D (2018). A role for calcium in resistin transcriptional activation in diabetic hearts. Sci Rep.

[59] Li Y, He Q, He CY, Cai C, Chen Z, Duan JZ (2023). Activating transcription factor 4 drives the progression of diabetic cardiac fibrosis. ESC Heart Fail.

[60] Zhang N, Yu H, Liu T, Zhou Z, Feng B, Wang Y, Qian Z, Hou X, Zou J (2023). Bmal1 downregulation leads to diabetic cardiomyopathy by promoting Bcl2/IP3R-mediated mitochondrial ca(2+) overload. Redox Biol.

[61] Gambardella J, Jankauskas SS, Kansakar U, Varzideh F, Avvisato R, Prevete N, Sidoli S, Mone P, Wang X, Lombardi A (2023). Ketone Bodies Rescue Mitochondrial dysfunction Via Epigenetic Remodeling. JACC Basic Transl Sci.

