# Supplementary figures and images for: Single-cell insights: pioneering an integrated atlas of chromatin accessibility and transcriptomic landscapes in diabetic cardiomyopathy
Source: Cardiovasc Diabetol. 2024 Apr 25;23:139. doi: 10.1186/s12933-024-02233-y (PMC11046823; doi:10.1186/s12933-024-02233-y)

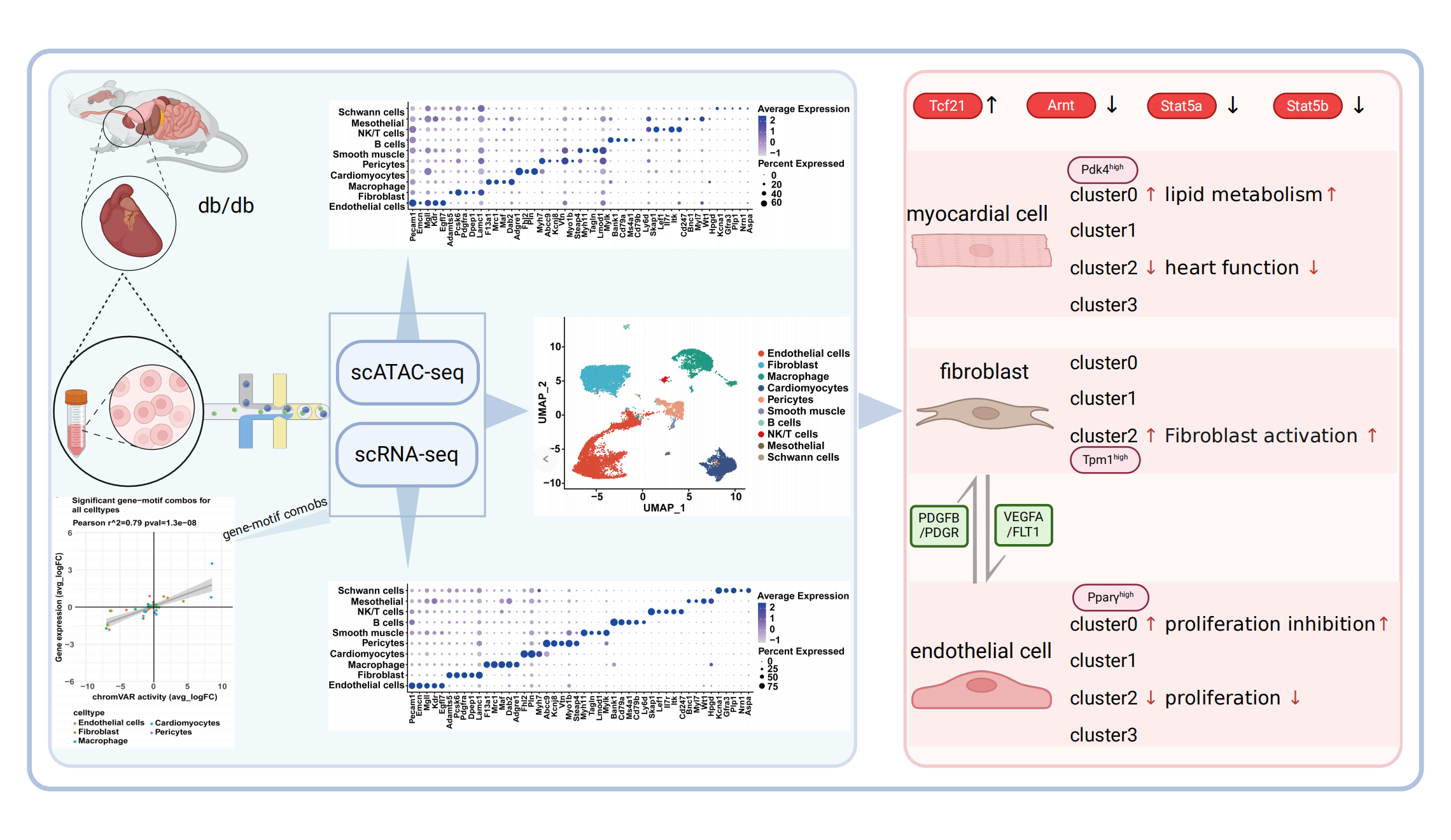

Supplement: Supplementary file 3 — Supplementary Material 3 [file 12933_2024_2233_MOESM3_ESM.tif]
