# Supplementary material for: Single-cell insights: pioneering an integrated atlas of chromatin accessibility and transcriptomic landscapes in diabetic cardiomyopathy
Source: Cardiovasc Diabetol. 2024 Apr 25;23:139. doi: 10.1186/s12933-024-02233-y (PMC11046823; doi:10.1186/s12933-024-02233-y)
Supplement: Supplementary file 4 — Supplementary Material 4 [file 12933_2024_2233_MOESM4_ESM.docx]

**Supplementary Information**


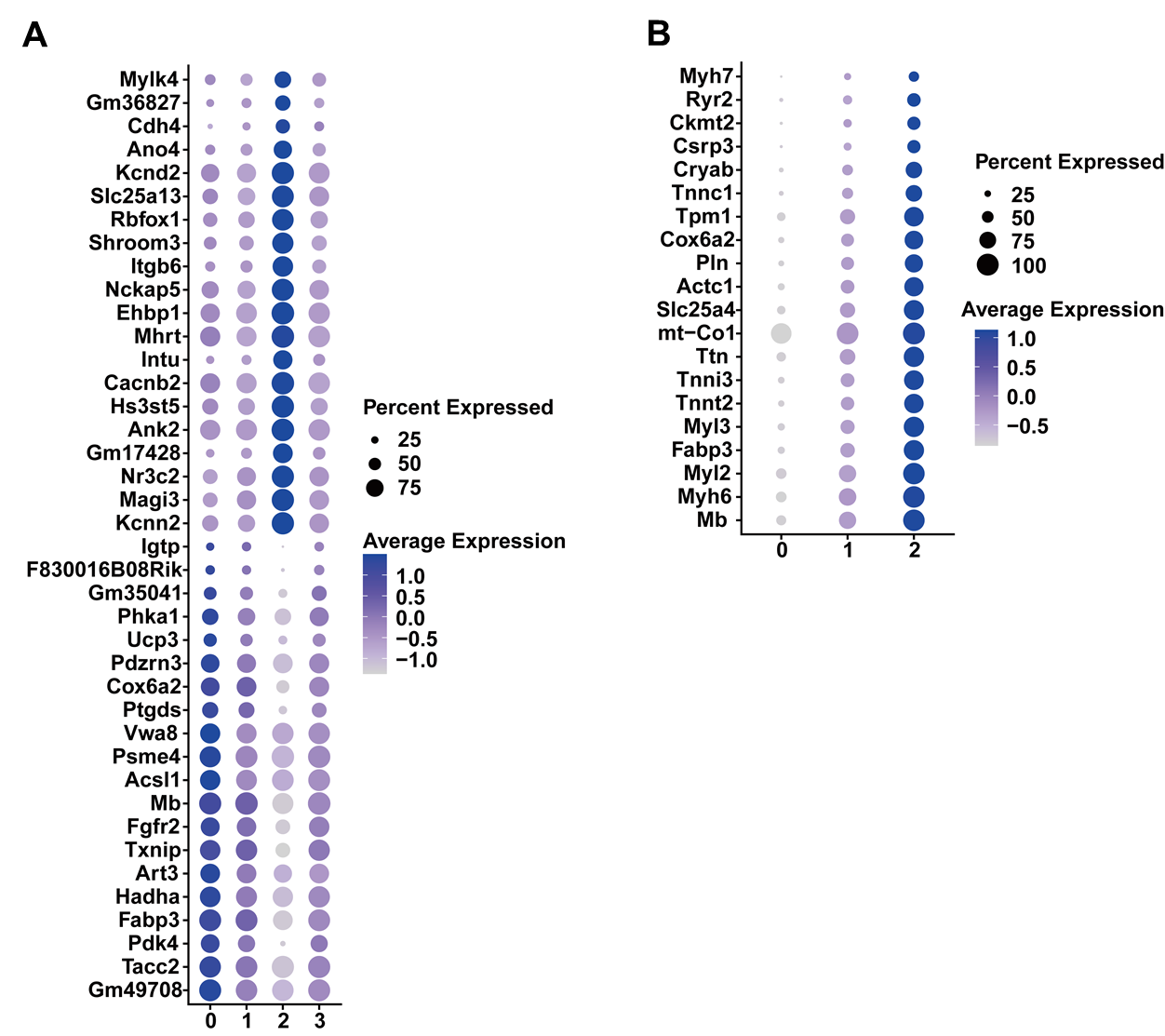


Supplementary Figure 1. Cluster-specific marker genes of cardiomyocytes and fibroblasts.

1. Bubble plot of cluster-specific marker genes of cardiomyocytes. The diameter of the dot represents the proportion of cells that express the corresponding gene and the density of the dot represents the average gene expression level among all subpopulations.

B. Bubble plot of cluster-specific marker genes of fibroblasts. The diameter of the dot represents the proportion of cells that express the corresponding gene and the density of the dot represents the average gene expression level among all subpopulations.

Supplementary Figure 2. Bubble plot of cluster-specific marker genes in endothelial cells. The diameter of the dot represents the proportion of cells that express the corresponding gene, and the density of the dot represents the average gene expression level among all subpopulations.
